# Ecosystem-specific microbiota and microbiome databases in the era of big data

**DOI:** 10.1186/s40793-022-00433-1

**Published:** 2022-07-16

**Authors:** Victor Lobanov, Angélique Gobet, Alyssa Joyce

**Affiliations:** 1 Department of Marine Sciences, University of Gothenburg, Box 461, 405 30 Gothenburg, Sweden; 2 MARBEC, Univ Montpellier, CNRS, Ifremer, IRD, Sète, France

**Keywords:** Community ecology, Meta-omics, Ecosystem-specific database, Data curation, Database management, Microbiota, Microbiome

## Abstract

The rapid development of sequencing methods over the past decades has accelerated both the potential scope and depth of microbiota and microbiome studies. Recent developments in the field have been marked by an expansion away from purely categorical studies towards a greater investigation of community functionality. As in-depth genomic and environmental coverage is often distributed unequally across major taxa and ecosystems, it can be difficult to identify or substantiate relationships within microbial communities. Generic databases containing datasets from diverse ecosystems have opened a new era of data accessibility despite costs in terms of data quality and heterogeneity. This challenge is readily embodied in the integration of meta-omics data alongside habitat-specific standards which help contextualise datasets both in terms of sample processing and background within the ecosystem. A special case of large genomic repositories, ecosystem-specific databases (ES-DB’s), have emerged to consolidate and better standardise sample processing and analysis protocols around individual ecosystems under study, allowing independent studies to produce comparable datasets. Here, we provide a comprehensive review of this emerging tool for microbial community analysis in relation to current trends in the field. We focus on the factors leading to the formation of ES-DB’s, their comparison to traditional microbial databases, the potential for ES-DB integration with meta-omics platforms, as well as inherent limitations in the applicability of ES-DB’s.

## Introduction

Interest in categorizing microbial communities across accessible habitats has exposed the vast complexity of microbial life [1–3]. What started with the laboratory isolation of microbial species from habitats of interest has expanded both in scope and depth following the advent of meta-omics (metabarcoding, metagenomics, metatranscriptomics, metaproteomics, metabolomics). Metabarcoding, for example, is now commonplace, allowing for an unprecedented systematic cataloguing of microorganisms using identifying biomarkers [4–6]. Technological developments over the past couple of decades have greatly expanded microbial community ecology analyses to include, albeit still at great cost and effort, the sequencing of all genomes within a sample (metagenomics). These deep dives into the microbial community allow a higher level of taxonomic precision as well as further opportunities to assess the functional capacity of the system [7–10]. Coupled to this has been an expansion of gene expression studies across community constituents within a sample (metatranscriptomics). The widening scope of meta-omics has led to the integration of diverse analytical tools into community ecology studies, such as metaproteomics and metabolomics, providing information on the underlying functional activity and metabolic state of the community, respectively [11, 12]. Measurements of physicochemical parameters (i.e., pH, EC, E_h_, temperature, nutrients) provide an environmental context for taxonomic and functional fluctuations within the microbial community. Integrating measures of microbial functionality with taxonomic identification and these contextual parameters is essential for a better understanding of inter-microbial relationships and their roles in a particular environment. Nonetheless, databases have largely catalogued their constituent datasets around data type (e.g., sequence data, physiological data) and not the environments from which organisms are being sampled. This practice results in less standardisation across studies utilizing different investigative strategies (i.e., different meta-omics approaches) on the same habitat, ultimately hindering the integration of multiple data types in microbial community ecology assessments.

Several studies have highlighted concerns over the validity of sequencing data accruing from the ever-expanding body of microbial surveys and microbiome studies [13–16]. One group of reviews has addressed this issue by proposing standards for studies to follow. These reviews target standardisation in the collection and processing of data for microbiome studies with respect to general guidelines [14, 17–19] and to specific environmental situations [20–25]. Another group of reviews has focused on the efforts to integrate other data types (e.g., mass-spectroscopy spectra, environmental physicochemical data) into sequencing studies [26–31]. These efforts notwithstanding, the evolution of microbial database collections from a data type orientation to an environment-specific one has received less attention. A recent commentary in Nature Microbiology addressed the topic of data type integration from the perspective of “microbiome centres”—institutions or consortia designed to accelerate microbiome research by facilitating collaborations between personnel and infrastructure resources [32]. While the inception of the Microbiome Centers Consortium (MCC; http://microbiomecenters.org/) in 2019 marks a milestone for more coordinated standardisation across microbiome studies, database resources are still developed largely independent of one another despite greater connectivity between laboratories around the world. Widespread use of diverse meta-omics techniques over the recent decades drives current efforts to streamline and integrate data types. Better database management achieves multiple aims: expanded access at an assured quality level, a repository for data, as well as more consistent and aligned standardisation for generating data. In this article, we review the development of ecosystem-specific databases (ES-DB’s) to address the unique challenges that arise when working with heterogenous data types inherent to microbial community ecology.

## Meta-omics tools to unravel microbial community diversity and function

**Fig. 1:**
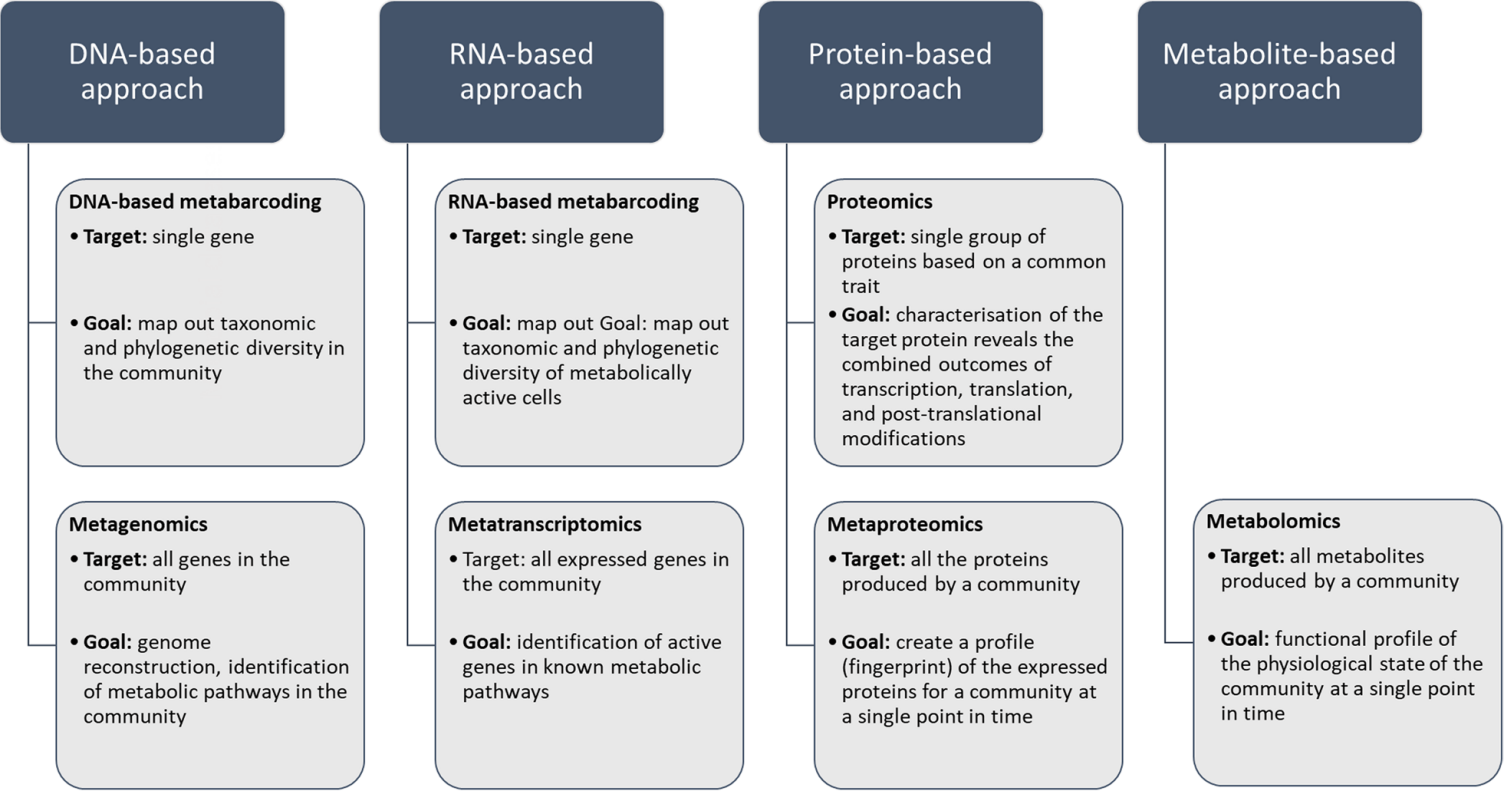
Interrelationships between multiple depths of biome characterisation, all which can be unified through microbial database collections

To study microbial community ecology, approaches used may be either DNA-based to study taxonomic diversity (metabarcoding) and gene diversity (metagenomics), or RNA-based to study gene-expression in the active microbial community (metatranscriptomics), or protein- and metabolite-based to study the production and secretion of various molecules (metaproteomics, metabolomics) (Fig. 1). Viruses, while not the primary focus of this review, are studied using both metagenomic and metatranscriptomics techniques, depending on the virus type targeted. Meta-omics tools are often used independently or a couple of them together in an effort to unravel complex interspecies relationships and functions in microbial communities. However, the lack of homogeneity among isolated studies limits their usefulness for making correlations and deriving meaningful hypotheses from similar studies.

### Taxonomic identification and diversity of the microbial community

Metabarcoding studies have largely succeeded in surveying microbial diversity in all major Earth habitats [33–37]. Several synonymous terms in widespread circulation include metataxonomics, community profiling, and amplicon sequencing. While metataxonomics and community profiling emphasise the putative categorical endpoint, amplicon sequencing highlights the methodological contrast to metagenomics. Metabarcoding may be done alone (sequences compared to reference databases) or against a metagenomic sample (sequences compared to dataset of the community genomes in a sample) [38–40]. The ability of high-throughput sequencing platforms to rapidly and accurately sequence gene regions has made metabarcoding data the most common data type in microbial sequence database collections [41–43]. Practical considerations such as a lower associated cost, lower DNA quantities and lighter bioinformatic analyses required per analysis further lend to the attractiveness of the method [44, 45]. The selection of universal gene sequences for each taxonomic rank of interest has been essential for yielding more exhaustive descriptions of microbial taxonomic diversity [46–49]. The increasing fidelity of third generation technologies such as the Pacific Bioscience and Nanopore long-read sequencing technologies and accompanying genome assembly infrastructure able to correct misreads represents an important milestone, allowing studies to rely on metabarcoding for species-rank taxonomic assignment [50–52].

Far from being a complete story, major biases persist around the metabarcoding approach as reviewed elsewhere [53–56]. Here we will emphasise the issue of unspecific amplification as a pernicious problem regardless of the focus on DNA- or RNA-based studies. For example, the use of the 16S rRNA gene to target bacteria also amplifies the 16S rRNA gene of plastids (e.g., chloroplasts, mitochondria) especially in host-associated microbial studies [57]. Similarly, investigations into host-associated microbial eukaryote interactions—typically targeting the 18S rRNA gene—simultaneously amplify host 18 s rRNA sequences. The use of excluding or blocking primers may mitigate these issues [58], however the challenges they pose have not been fully resolved.

The requirement for amplification of a known sequence sets metabarcoding apart from other genetic investigation methods and is a significant limitation in exploratory research. For taxonomic studies, targeted genes must be variable enough to distinguish species or strains yet have sufficiently conserved sequences flanking the gene of interest in order to design primers. Common standards include the 16S rRNA gene for prokaryotes [59–62], the 18S rRNA gene (microbial eukaryotes) [63], or the ITS region (fungi) [64, 65], albeit other sequences such as heat shock proteins have promising perspectives [66, 67]. Additionally, while metabarcoding typically involves sequencing the small subunit of the rRNA gene, it may also be applied to other genes of interest [68, 69]. By identifying the presence of a gene, these procedures are able to provide a clue into the metabolism exhibited by the sampled organism.

For studies aiming only to describe community composition, metabarcoding remains a cost-effective tool compared to culture-independent techniques- especially for well-studied microbiomes [33, 61, 70]. The most significant shortcoming in metabarcoding is the limited flexibility of a single gene sequence to represent total diversity. In contrast to the sequencing of a specific amplicon, targeted metagenomics is a culture-independent technique which limits the scope to a subset of total sample diversity (e.g., prokaryotes or eukaryotes) but targeting all genes within the sample [71, 72]. Ultimately, while sequence data is effective at mapping taxonomic relationships within a sample, investigative work into the mechanisms driving observed shifts in physicochemical parameters require a broader set of tools. Meta-omics addresses this goal by exploring community ecology from different perspectives: what microorganisms may potentially create (metagenomics), what they are in the process of creating (metatranscriptomics), and what they have created (metaproteomics, metabolomics).

### Function and metabolic potential of the microbial community

Discerning function within the context of microbial ecosystems is a major challenge for community ecology studies. Metagenomic studies reveal functional potential—DNA sequences that have the potential to be expressed. Conversely, metatranscriptomics can be used to study the pool of expressed sequences. The subset of expressed sequences may then be studied within the context of translated proteins (metaproteomics) or as the biproducts of cellular metabolism (metabolomics). The following section will describe the types of data produced in these studies as well as the unique challenges they pose in terms of database integration.

#### Metagenomics

The metagenomic approach indiscriminately sequences all DNA fragments from a sample. The goal of this approach is to preserve the vast majority of genomes, with the important caveat that sampling the true total diversity is not physically realistic [73, 74]. Metagenomics is not subject to the same limitations in terms of primer selection and specificity as metabarcoding [45, 75], although data quality may be diminished through pre-processing steps as with other methods [53, 54, 76]. Given the significantly larger datasets than found in metabarcoding studies, metagenomics data is more laborious to process with requirements for sequence preparation and assembly that must be weighed against the potential for greater resolution in discerning metabolic pathways [77]. In assembly, short sequencing reads are sorted to link extracted genomes with the original mixed microbial community constituents. The resulting pool of genomes can be screened for the presence of genes associated with metabolic pathways of interest; however it does not confirm their expression (requiring transcriptomics).

The assembly of DNA-based viruses was initially beset with unique challenges compared to other sources of DNA, however the isolation and sequencing of DNA-based viruses has become considerably more developed and reproducible, as reviewed elsewhere [78–80]. Similarly, RNA-based viruses have also been studied through metagenomics, with protocols now achieving a high degree of accuracy and recovery efficiency [81].

Indeed, some pathways may be shared among several genomes, suggesting potential cross-feeding between microbes in the community [82]. Screening genomes for specific genes associated with particular metabolic profiles can be a powerful tool in discerning (i) evolutionary acquisition of genes and (ii) putative biochemical transformations within the ecosystem. Such information may help substantiate observed physicochemical shifts in the ecosystem. Crucially, however, metagenomic sequence data from reference databases cannot linearly translate into functional assignments for homologous sequences [15, 83], in contrast to metatranscriptomics which measures gene expression (84, 85).

#### Metatranscriptomics

The application of metatranscriptomics to analyse the sum of genes expressed in a same sample, is essential for assigning functionality to members of a microbial community. The metatranscriptomics approach explores the metabolically active fraction of a sample via sequencing RNA transcripts. Here, total RNA or messenger RNA (mRNA) in a sample is converted to complementary DNA (cDNA), allowing it to be further pre-processed as needed in a stable form [86, 87]. The cDNA strands are then sequenced, creating a map of active gene expression and regulation [88, 89]. Analogous to DNA-based approaches, metatranscriptomics can be PCR-mediated or PCR-free, furthermore, reads may be assembled de novo or with the help of a reference database [90–92]. The most challenging aspect of metatranscriptomics is the isolation and storage of microbial mRNA as mRNA must be separated from rRNA, since the latter comprises the majority of RNA present, as well as from eukaryotic mRNA [87, 93, 94]. Furthermore, the inherent instability of mRNA reduces the amount of sample available for sequencing [94]. Strategies whereby RNA is stabilised (e.g., poly(A) tailing) limit the need for sequence knowledge prior to cDNA synthesis [95–97]. While this improves on previous primer-based sequencing methods, it nonetheless presents new biases and challenges [88, 98–100]. RNA- viruses have been characterized through metatranscriptomics [101, 102]. Limitations in the bioinformatics infrastructure for reference databases as well as the quality of submissions nonetheless hampers the ability to work with viral strains [93], albeit the issue is being addressed by organisations such as the World Society for Virology [103]. As the case with virtually all metagenome reference datasets, transcriptome reference databases suffer from significant coverage gaps [92]. Furthermore, the task of relating transcriptomic data to DNA-based taxonomy presents its own set of challenges [104].

#### Metaproteomics

Instead of focusing exclusively on protein diversity within a sample, metaproteomics provides a temporo-spatial snapshot of the proteins expressed by the metabolically active community [105, 106]. Metaproteomics includes all analyses that isolate or characterise proteins, such as two-dimensional gel electrophoresis, liquid chromatography, mass spectrometry, as well as antibody and protein microarray techniques [107]. Limitations to metaproteomic approaches are typically associated with the complexity of the sample, especially in dynamic environments with multiple trophic levels [108, 109]. The current state of the field and the challenges within have been reviewed elsewhere [110–113].

Proteins from animals, plants, or otherwise non-target organisms often contribute to samples, and that further complicates already sensitive protein extraction methods [114]. Finally, researchers must contend with computational challenges in the identification and assignment of peptides and proteins [115–117] as well as their functional annotation [118].

#### Metabolomics

Metabolomics seeks to identify and quantify metabolites (compounds ≤ 1500 Da) produced by the metabolically active fraction of the microbial community [105]. Generally, metabolomics is most effective when investigating how a known set of metabolites produced by a particular community may change under experimental conditions. It is particularly effective as a tool to follow the response of organisms to changes in stimuli (nutrition, biotic and abiotic stressors) [119–121]. Since changes in the metabolome occur simultaneously with changes in the transcriptome and proteome, mapping of biochemical pathways can theoretically link metabolomics with both -omics results. However, there are practical challenges around handling large amounts of metabolomics data as well amplicon and protein data when there can also be insufficient reference data and difficulties in profiling metabolites. As such, metabolomics is typically split between metabolite profiling (labor-intensive but specific) and fingerprinting (rapid but only partially correlatable snapshot) [122]. Compared to metaproteomics, metabolomics faces greater challenges owing to the colossal quantity of metabolites present in any sample and the large number of uncharacterised metabolites [123]. Furthermore, accurately detecting molecules is limited by detection methods (e.g., interpreting MS peaks) as well as the detection of partially degraded metabolites—both factors contributing to false discoveries [124]. A major asset in unravelling metabolic pathways has been the emergence of constraint-based analyses of metabolic networks, which are able to integrate gathered data with simulated metabolic models. Of these, the most predominant is the flux balance analysis (FBA) which accompanies mechanistic simulations with the stoichiometric matrices for the conservation of mass and biologically relevant objective functions to predict flux distributions. These networks may then be further explored through metabolic pathway analysis, which creates potential pathways between sets of metabolites. A common thread in the analysis of biological data is the excess of variables, lending to a potential over-reliance on reference databases or established models. Developments such as the Metabolomics Standards Initiative support the creation of more reliable protocols to determine whether metabolites of interest are present in samples, while projects such as the Human Metabolome Database create a more specialised reference tool for human studies [125, 126].

#### Functional assignment

While elucidating function is a major goal of meta-omics, biochemical observations cannot be directly mapped back onto sequence data. Even though several strategies exist to segregate the metabolically active microbial community from the total of detectable genetic sequences [127, 128], a similar strategy does not yet exist to segregate the total exudates predicted by the metatranscriptome from those observed through metabolomic or metaproteomic analyses. Nonetheless, there are several initiatives that incorporate ontological analysis with the large sequence datasets generated from metagenomic studies, which is an area that has been well reviewed [18, 129–131]. Ultimately, this is due to a diverse set of challenges: full or partial degradation of exudates before sampling, modification of compounds (e.g. use as reducing equivalents), inadequate sampling resolution, etc. There is a plethora of physiological observations of isolated strains in the laboratory and putative inferences provided by metagenomic analysis, but community ecology aims to describe on a physicochemical level the potential of a microbial community to interact within its ecosystem [127, 132–135]. For more information on the state of meta-omics for functional community analysis, the reader is referred to recent studies [26, 29, 30, 136–140].

## Common microbial databases

The accumulation of data in large taxonomic repositories has opened up new possibilities for research into the organisation and assembly of microbial communities that were previously inaccessible due to sparse coverage. Persistent incomparability of microbiome analyses has been addressed by consolidating studies around the same set of metadata standards [141, 142]. Given the rapid proliferation of heterogenous data generated from different protocols with and without standardisation steps, several prominent institutions have set out to create more internally consistent generic repositories for datasets. A selection of prominent databases are summarised in Table 1, while a more thorough and regularly updated list can be found in the annual issue of *Nucleic Acids Research* devoted to databases [143].

**Table 1:** Examples of public databases for microbial community analysis. Prevalent microbial sequence databases are listed below with indications of their omics integration and functional assignment integration where applicable

Database name	Data type	Meta-omics approach included	Target organisms	URL	References
China National GeneBank (CNGB)	rRNA subunits Genomes Transcriptomes Proteomes Environmental/ contextual data	Sanger sequencing Metabarcoding Metagenomics Metatranscriptomics Metaproteomics Environmental measurements	All microorganisms	https://db.cngb.org/	[185]
ConsensusPathDB	rRNA subunits Genomes Transcriptomes Proteomes Environmental/ contextual data	Sanger sequencing Metabarcoding Metagenomics Metatranscriptomics Metaproteomics	Animal (human, mouse), fungi (yeast)	http://consensuspathdb.org/	[186, 187]
DNA DataBank of Japan (DDBJ)	rRNA subunits Genomes Transcriptomes Proteomes Environmental/ contextual data	Sanger sequencing Metabarcoding Metagenomics Metatranscriptomics Metaproteomics	All microorganisms	http://www.ddbj.nig.ac.jp	[188–190]
European Molecular Biology Laboratory—European Bioinformatics Institute (EMBL-EBI) European Life-Science Infrastructure (ELIXIR)	rRNA subunits Genomes Transcriptomes Proteomes Metabolomes Environmental/ contextual data	Sanger sequencing Metabarcoding Metagenomics Metatranscriptomics Metaproteomics Metabolomics Environmental measurements	All microorganisms	https://elixir-europe.org/	[191–197]
EzBioCloud	rRNA subunits Genomes Environmental/ contextual data	Sanger sequencing Metabarcoding Metagenomics Environmental measurements	Bacteria and Archaea	https://www.ezbiocloud.net	[198]
International Nucleotide Sequence Database Collaboration (INSDC)	rRNA subunits Genomes	Sanger sequencing Metabarcoding Metagenomics	All microorganisms	https://www.insdc.org/	[199, 200]
Joint Genomic Institute Integrated Microbial Genomes (JGI- IMG)	rRNA subunits Genomes Transcriptomes Proteomes	Sanger sequencing Metabarcoding Metagenomics Metatranscriptomics Metaproteomics	All microorganisms	https://img.jgi.doe.gov/index.html	[201]
Metagenomic Rapid Annotations using Subsystems Technology (MG-RAST)	rRNA subunits Genomes Transcriptomes	Sanger sequencing Metabarcoding Metagenomics Metatranscriptomics	All microorganisms	https://www.mg-rast.org/	[202, 203]
National Center for Biotechnology Information collections (NCBI RefSeq, NCBI BLAST, NCBI Entrez, NCBI GenBank)	rRNA subunits Genomes Transcriptomes Proteomes Metabolomes Environmental/ contextual data	Sanger sequencing Metabarcoding Metagenomics Metatranscriptomics Metaproteomics Metabolomics Environmental measurements	All microorganisms	https://www.ncbi.nlm.nih.gov/refseq/ https://www.ncbi.nlm.nih.gov/refseq/ https://www.ncbi.nlm.nih.gov/search/ https://www.ncbi.nlm.nih.gov/genbank/	[204–207]
Protist ribosomal reference database (PR2)	rRNA subunits	Sanger sequencing Metabarcoding	All eukaryotes	https://pr2-database.org/	[208]
SILVA	rRNA subunits	Sanger sequencing Metabarcoding	All microorganisms	https://www.arb-silva.de/	[209]
University of California, Santa Cruz Genome Browser	rRNA subunits Genomes Transcriptomes	Sanger sequencing Metabarcoding Metagenomics	All microorganisms	https://genome.ucsc.edu/	[210]
Ribosomal RNA operon copy number database (rrnDB)	rRNA subunits	Sanger sequencing Metabarcoding	Bacteria and Archaea	https://rrndb.umms.med.umich.edu/	[211]
The Microbe Directory (TMD)	rRNA subunits Genomes Environmental/ contextual data	Sanger sequencing Metabarcoding Metagenomics Environmental measurements	Microbial prokaryotes and eukaryotes	https://coda.io/@themicrobedirectory/home	[212]
Vienna Metabolomics Center (VIME)	rRNA subunits Genomes Transcriptomes Proteomes Metabolomes	Sanger sequencing Metabarcoding Metatranscriptomics Metaproteomics Metabolomics	All microorganisms	https://vienna-metabolomics-center.at/	[213]

A fundamental challenge for the collection of microbial community data is the unequal incorporation and treatment of experimental parameters across studies. Biases in data collection, processing, and interpretation are not necessarily controllable or due to human error. Environmental and technological constraints, as well as the inherent need to choose different sampling techniques, hamper reproducibility across studies. The inception of “microbiome centers” as a knowledge sharing network that seeks to promote cross-disciplinary integration and to streamline the collection and analyses of microbial community data is an emergent strategy to respond to this challenge [32].

The microbial database collections described in Table 1 share a fundamental characteristic; they specialise in specific data types and targeted taxa rather than ecosystems. By contrast, ecosystem-specific databases adapt methodologies and analyses to the unique characteristics of the ecosystem under study. Nonetheless, ecosystem-specific databases are still an emerging tool for a better understanding microbial community dynamics. The following sections cover the motivations leading to their emergence, as well as an analysis of their benefits and limitations in describing microbial community ecology. 

## Ecosystem-specific databases as a platform for standardisation of methodological practices

A decoupled approach, whereby each -omics study presents a subset of the entire picture, addresses some of the inherent practical limitations (cost, expertise, infrastructure requirements) behind multi-omics studies. Common sets of standards are essential for integrating temporally and spatially distinct studies of the same ecosystem into a coherent view of the whole, and in this respect, ecosystem-specific databases provide a useful template [144]. In turn, this likely contributes to the generation of higher quality results in terms of accuracy, precision, and reproducibility. Common standards across studies facilitate the integration of meta-omics tools into community analysis, as well as the ability for multiple studies to be included as additional temporal and spatial snapshots of a sampling region. Furthermore, ES-DB’s are always curated by a research group or consortium with experts of the given ecosystem and/or targeted taxa. While the inclusion of metadata greatly improves the quality of microbial databases [14, 145–148], reliably identifying errors (mislabeled data, misspelled labels [149]) within large data sets remains a challenge [150–152].

An inherent advantage of ES-DB’s is improving interconnectivity of studies around the same ecosystem. Multiple studies describe the usefulness of manual curation to complement automated assignment tools [153–156]. As a case study for the statistical power of combining studies into aggregate databases through standardised methodologies, the Earth Microbiome Project Consortium (EMP) collected and analysed data from 97 microbiome studies, 59 of which were published in peer-reviewed journals [157]. Owing to standardised protocols, pooled data from the EMP has been used in meta-analyses to contextualise global patterns derived from individual studies [158]. Within the EMP consortium, individual studies are able to tailor collection and analysis practices to their unique environment under study while adhering to a set of core standards. As such, it represents an example of how both standardization and customization may be weaved together.

Curated databases have allowed for development of sampling protocols tailored to the microbial communities under study. One such example is the *Actinobacteria* genus, *Tetrasphaera*, that was routinely underestimated in wastewater treatment systems until microbial screening protocols incorporated adaptations to the cell lysis procedure during DNA extraction [159]. These procedural adaptations, concomitant with a push for greater reproducibility across microbial community studies investigating wastewater treatment, have contributed to the formation of the Microbial Database for Activated Sludge (MIDAS 3). MIDAS has since become a detailed ecosystem-specific database for wastewater treatment systems with resolution at the species level [160, 161]. Since then, the MIDAS team has also included a field guide for researchers interested in submitting their own data [162].

A critical aspect of database management is the development of internal quality standards. What has been described as the reproducibility crisis, a phenomenon whereby microbiome studies often produce poorly comparable datasets and interpretations, may be addressed through the standardisation of methodologies and interconnectivity among researchers [53, 163–165]. Each step in the analysis of microbiomes will influence the resulting OTU table from sample storage, DNA extraction, sequencing (including as applicable: amplification, primer choice), sequencing platform, to the choice in bioinformatics pipeline used. As such, while standardisation does not remove biases involved in the process, it may reduce variability across studies in the same field. A recent review on the critical knowledge gap around sampling and handling in microbiome studies identified 95% of studies as having used subjective sampling methods or inadequately describing a methodology [76]. Schloss (2018) recently outlined how microbiome studies can improve their integrity and reproducibility through an evaluative rubric [163]. Data transparency has likewise been shown to improve community cross-validation [16, 166, 167]. The standardisation of bioinformatics processes has been facilitated by independent, community-led initiatives such as the Critical Assessment of Metagenome Interpretation (CAMI), a comprehensive comparison of methodologies for microbiome analysis [168]. Other sources provide more general guidelines and educational tools such as the Statistical Diversity Lab (http://statisticaldiversitylab.com/) [169], as well as resources that summarise best practices in sample preparation for microbiome analyses [14, 170]. In contrast to the above protocols that present ways in which standardisation can be done, ES-DBs establish standards in the context of their specific biome. Table 2 summarises current ES-DB’s in operation and their capabilities.

**Table 2:** A selection of published ecosystem-specific databases

Ecosystem-specific database	Target ecosystem(s)	Target organisms	Meta-omics approach used	References
Biomes of Australian Soil Environments (BASE)	Australian subcontinent, terrestrial systems	Prokaryotes and fungal-specific eukaryotes	Sanger sequencing Metabarcoding Metagenomics Environmental measurements	[214]
Dictyopteran gut microbiota reference Database (DictDb)	Dictyopteran gut microbiota	All microorganisms	Sanger sequencing Metabarcoding Metagenomics	[215]
Earth Microbiome Project (EMP)	EMP Ontology (EMPO) ecosystems	All microorganisms	Sanger sequencing Metabarcoding Metagenomics Metatranscriptomics Metaproteomics Metabolomics Environmental measurements	[34, 216]
Genome Repository of Oiled Systems (GROS)	Crude oil contaminated environments	All microorganisms	Sanger sequencing Metabarcoding Metagenomics Metatranscriptomics Environmental measurements	[217]
Global Ocean Sampling (GOS)	Open ocean ecosystems	All microorganisms	Sanger sequencing Metabarcoding Metagenomics Metatranscriptomics Metaproteomics Metabolomics Environmental measurements	[218]
Human Food Project	Human gastrointestinal tract	All prokaryotes	Sanger sequencing Metabarcoding Metagenomics Metatranscriptomics Metaproteomics Metabolomics Environmental measurements	[219]
Integrative Human Microbiome Project (HMP)	Human body microbiome environments	All microorganisms	Sanger sequencing Metabarcoding Metagenomics Metatranscriptomics Metaproteomics Metabolomics Environmental measurements	[181–183]
Human Oral Microbiome Database (HOMD)	Human oral environment	All microorganisms	Sanger sequencing Metabarcoding Metagenomics Metatranscriptomics Metaproteomics Metabolomics	[220]
Maarja Öpik arbuscular mycorrhiza database (MaarjAM)	Arbuscular mycorrhizal fungi associated environments	All microorganisms	Sanger sequencing Metabarcoding Metagenomics Environmental measurements	[221]
Marine databases; MarRef, MarDB, MarCat	Open ocean ecosystems	All microorganisms	Sanger sequencing Metabarcoding Metagenomics Metatranscriptomics Metaproteomics Metabolomics Environmental measurements	[184]
METAgenomics of the Human Intestinal Tract (MetaHIT)	Human gastrointestinal tract	All microorganisms	Metagenomics Metatranscriptomics Metaproteomics Metabolomics	[222]
Microbial Database for Activated Sludge (MiDAS)	Activated sludge	All microorganisms	Sanger sequencing Metabarcoding Metagenomics Metabolomics Environmental measurements	[223]
Rumen and Intestinal Methanogen- DB (RIM-DB)	Ruminant gastrointestinal tract	All microorganisms	Sanger sequencing Metabarcoding Metagenomics	[224]
Tara Oceans project	Open ocean ecosystems	All microorganisms	Sanger sequencing Metabarcoding Metagenomics Metatranscriptomics Metaproteomics Metabolomics Environmental measurements	[35, 225, 226]
Unified Human Gastrointestinal Genome (UHGG) collection	Human gut	All microorganisms	Sanger sequencing Metabarcoding Metagenomics Metaproteomics	[227]

### A roadmap for ecosystem-specific databases

Environment-specific databases typically originate around persistent knowledge gaps and are often associated with challenges in the selection of appropriate sampling techniques. This is the case of the proposed Drinking Water Microbiome Project (DWMP) outlining a knowledge gap from a literature comparison that indicated a lack of knowledge within the drinking water microbiome literature compared to other wastewater treatment microbiomes [171]. They propose that a common database allowing diverse data types to be pooled under standardised conditions can address the challenge of characterizing microbiome dynamics for drinking water systems. A recent perspective article by de Vrieze (2020) discussed the creation of a more applied database than currently available within the MIDAS infrastructure to address the challenges in studying the anaerobic digester microbiome [172, 173]. Here, a strategy of identifying and fingerprinting microbial communities within the anaerobic digestion microbiome is proposed as a tool to complement monitored physicochemical parameters. As measurements reveal shifts in the concentration of specific metabolites, this may be related to shifts in the community composition at large.

In all cases, the integration of functional databases with taxonomic collections requires both top-down and bottom-up engagement as proposed for the DWMP [171]. A recent meta-analysis of DNA barcoding databases that cover European aquatic habitats highlighted issues in quality control and assurance when integrating diverse databases; results pointed to an inconsistent image of taxonomic and subsequently phylogenetic diversity [174]. Despite this, interest in greater biome contextualisation as well as cross-biome studies appears to be growing. A consortium of researchers studying water quality in natural and anthropogenic environments, the Alliance for Freshwater Life, demonstrates how properly curated and inclusive databases may communicate with a larger audience and develop policy and educational platforms beyond their fundamental scientific contribution [175]. Importantly, ecosystem-specific datasets are not limited to environmental studies. In their 2018 article, Kapono et al. recreated the “human environment” as a combination of microbial and chemical data for use in forensics studies [176]. Nor has the applicability of identifying microbiome-associated biomarkers or keystone species been ignored in health and medicine [170, 177, 178]. Similarly, the search for novel genes via bioprospecting depends strongly on accurate genetic annotation and thus may also benefit from more robust reference databases [179, 180].

### Limitations of ES-DB’s for meta-omics integration

ES-DB’s appear to be well conceived to address some of the contemporary challenges associated with large microbial community datasets: standardisation of sample methods, processing and analysis, data reproducibility, and the integration of meta-omics technologies from independent studies on the same ecosystem, as reviewed previously [53, 165]. In essence, the goal of ES-DB’s is to ensure that anthropogenic biases (sampling strategies, analysis protocols) are kept to a minimum so that (i) temporal and spatial variability may be better studied across independent studies of the same ecosystem and (ii) independent research groups specializing in different meta-omics analytical strategies are all able to contribute towards a common knowledge pool.

Pinning down an explicit definition for ecosystem-specific databases in contrast to multi-omics databases can become blurred, since it depends on how the ecosystem in question is defined. While in some cases the ecosystem under study is physically constrained (e.g., human body microbiome [181–183]), in other cases it describes a global system (e.g., the open ocean [184]). Biomes do not have strict boundaries, so ES-DB’s may suffer from arbitrary exclusions of relevant data from neighboring biomes. Adding or subtracting biomes into the scope of a particular ES-DB will necessarily lead to blurring definitional boundaries and a form of the Sorites paradox, which pursued to its logical conclusion can eventually broaden an ES-DB into a generalised microbial collection. A grey area emerges when it comes to describing the boundary between databases examining multiple biomes within a common specialised environment and databases examining them within a global holistic context. Generic databases thus remain an effective catch-all option for any data type.

Another crucial limitation to ES-DB’s relates to their administration. In order to have professional curation of the dataset, there must be a group of specialists in the field willing and able to provide the service. One way in which the initial entry costs could be lowered would be to establish a standardised template (meta-structure), applicable to any microbial database collection, for data that is to be uploaded or pooled from existing datasets. This strategy could accommodate any dataset size that is collected by a single research group up to an international consortium, with curation rights regulated by each database founder. Not only would this allow better integration between ES-DB’s, but it could decrease the barriers to entry by removing the need for extensive bioinformatics expertise. It would provide a template for decision-making by researchers to follow with respect to sample processing and data organisation. Alongside the emergence of ES-DB’s, several “utilitarian databases” have been proposed that orient themselves around functional analyses, ecosystem services, and/or the organisation of metadata (Table 3). As the scope and depth of these auxiliary tools expands, they will further complement the development of databases and analytical tools catering to unique ecosystems.

**Table 3:** A non-exhaustive list of organisational databases pooling data from other sources as an analytical tool

Functional database	Purpose	Description	References
Functional Ontology Assignments for Metagenomes (FOAM)	Functional analysis	Groups environmental metagenomic sequences based on gene functionality instead of taxonomy	[228]
EXPath	Functional analysis	Groups microarray expression profiles used to infer metabolic pathways for six model plants	[229]
Ecopath with Ecosim (EWE) (now grouped under EcoBase)	Functional analysis	Information repository of EwE models (modeling software for ecological phenomena)	[230]
Gulf of Mexico Ecosystem Services Valuation Database (GecoServ) (now called BlueValue)	Ecosystem service evaluation	Worldwide depository of ecosystem valuation data	[231]
Open access database on climate change effects on littoral and oceanic ecosystems (OCLE)	Ecosystem service evaluation	Ecological-driven database of present and future hazards for European marine life	[232]
Biofuel Ecophysiological Traits and Yields Database (BETYdb)	Functional analysis	Open-access repository to facilitate the organisation, discovery, and exchange of information about plant traits, crop yields, and ecosystem functions	[233]
jae-f-database	Functional analysis	Global database and ‘state of the field’ review of research into ecosystem engineering by land animals	[234]
Genomes OnLine Database (GOLD)	Metadatabase	Collection of genome projects and associated metadata	[235]
Omics Discovery Index (OmicsDI)	Metadatabase	Groups datasets across multiple public meta-omics data resources	[236]
Omics database generator (ODG)	Metadatabase	Groups genomics data, integrates with experimental data to create a comparative, multi-dimensional graphical database	[237]

## Conclusion

The establishment of generic repositories for genetic data marked a milestone for the systematisation of global microbial diversity cataloguing. Having greatly expanded data accessibility, data type specific sequence and omics repositories facilitate novel analyses of data collected from previous studies. However, different standards and practices around data collection and processing reduce data robustness and limit the ability for researchers to compare studies [53, 165]. Although no generalizable model for standardisation can be applied across all ecosystems, standards applied to a restricted ecosystem can be useful. Here we have reviewed how various factors contribute to the emergence of ecosystem-specific databases and what important repercussions for data quality and reproducibility can emerge from well-considered strategies that integrate multiple data types.

Nonetheless, more widespread implementation of ES-DB’s requires more inclusive and accessible bioinformatic infrastructure. While algorithms and methodologies designed to sort and organise existing data are becoming more widespread, only a few resources are available to facilitate spontaneous creation of new ES-DB’s. Concrete standards for data annotation and organisation that permit better synthesis of omics data are necessary to facilitate this development. By consolidating standards for best practices and professionally curating data, higher quality and reproducible datasets will become more commonplace and accessible in the future.

A final point along these lines is that a good database requires good datasets. Standard methods are a representation of best-practices in a world of practical and economic limitations. As technology improves, database curators must decide when and how to update the standard methodology, taking into consideration that each shift damages the reproducibility of the database as a whole. As an ongoing example, significant reductions in the cost of full-length 16S rRNA gene sequencing are making longer reads increasing competitive strategy vs. shorter amplicons—the current recommended sequencing strategy for databases such as the EMP. Currently, the Illumina platform (specializing is short reads) delivers a higher sequencing quality than Pacific Bioscience and Nanopore (long reads)—a crucial decision factor which will also need to be resolved. The entry barrier for new data will need to be set individually across ES-DBs to balance expanding the breadth of incoming datasets against constricting data to only high-quality entries. Nonetheless, curation will only continue to rise in importance as database collections increase in both size and scope.

## Data Availability

Not applicable.

## Abbreviations

CAMI: Critical Assessment of Metagenome Interpretation
DWMP: Drinking Water Microbiome Project
EMP: Earth Microbiome Project
ES-DB: Ecosystem specific database
MCC: Microbiome Centers Consortium
MIDAS: Microbial Database for Activated Sludge

## Glossary

Biome: Total biotic diversity within a habitat
Community ecology: Identification of taxonomic and phylogenetic relationships between organisms in a community including how they react to their non-living surroundings.
Database: A collection of data arranged around specific characteristics making it easier for retrieval. Sequence databases contain digitalized representation of biological informational units (e.g., nucleic acids, proteins) however databases may include representations or descriptions for other types of biological data
Ecosystem: A biological community of interacting organisms and their physical environment
Functional analysis: Relating expressed genes or metabolites produced to taxonomic identity utilizing meta-omics data
Genomics: A field of study involving all aspects of genomes from their structure, evolution, as well as readability (mapping) and functionality
Habitat: Physical (abiotic) and biotic resources present in a particular area
Keystone taxon: A taxon having a disproportionate influence on community structure, where the influence is due to strong biotic interactions rather than high abundance
Meta-: A prefix indicating that the following term applies to a community sample (see metagenomics vis à vis genomics). The term “metabolomics” has a different root and does not follow this pattern
Metabarcoding: Method allowing for the identification of all the species in the community by targeting a specific gene or gene region
Metadata: All data describing the characteristics of the entry, how it is stored and defined
Metadatabase: A compilation of databases based on their metadata. This centralises screening, comparing, and filtering databases making the data more accessible
Metagenomics: Method targeting the amplification of all genes directly from an environmental sample
Meta-omics: An umbrella term encompassing genomics, transcriptomics, proteomics, and metabolomics. Sometimes referred to as multi-omics
Metaproteomics: A term encompassing all experimental approaches related to the study all proteins in microbial communities, generally their identification and quantification in complex samples
Metastructure: The underlying structure used to organise metadata
Metatranscriptomics: Method allowing for the identification of all expressed genes in an environmental sample
Microbiome: Refers to the total conceptual collection of microorganisms and their genomes within a specific environment
Microbiota: Refers to the total physical collection of microorganisms within a specific environment
-Omics: A suffix referring to a range of biological disciplines, often grouped together as a tool-kit for biological analyses of microbial communities: genomics, proteomics, metabolomics, metagenomics, phenomics and transcriptomics
Proteomics: The isolation and study of proteins from a single organism
Targeted metagenomics: Subset of metagenomics whereby subsequent sequencing analysis constricts the study focus, i.e., to a specific gene cluster or particular group of organisms
Transcriptomics: Analysis of gene expression (entire genome, single gene, or gene cluster) within a single organism
